# Factors Influencing the Choice of Conservative and Surgical Procedures in Dental Patients from Poland: A Single-Center Retrospective Analysis

**DOI:** 10.3390/jcm14051508

**Published:** 2025-02-24

**Authors:** Kacper Łaganowski, Martyna Ortarzewska, Kornela Cieślik, Jakub Jankowski, Kacper Nijakowski

**Affiliations:** 1 Department of Conservative Dentistry and Endodontics, Poznan University of Medical Sciences, 70 Bukowska Street, 60-812 Poznan, Poland; k.laganowski@ump.edu.pl (K.Ł.); mortarzewska@ump.edu.pl (M.O.); jjankowski41@wp.pl (J.J.); 2 University Center of Dentistry and Specialized Medicine, 70 Bukowska Street, 60-812 Poznan, Poland; k.cieslik@ucs.poznan.pl

**Keywords:** gender, tooth restoration, root canal treatment, dental surgery, age, decision making

## Abstract

**Background/Objectives**: Oral health behaviors can be shaped by various factors, both global (such as the COVID-19 pandemic) and individual (e.g., gender, age). This retrospective study aims to assess the factors influencing the choice between conservative and surgical dental procedures among patients based on the example of the university specialized center in Poznan. **Methods**: We explored the patient dataset from the University Center of Dentistry and Specialized Medicine (Poznan, Poland), covering the period from 1 January 2017 to 31 December 2023. A total of 182,654 medical records were analyzed, focusing on procedures such as restorations, endodontic interventions, and extractions. Multivariate logistic regression and multidimensional correspondence analyses were employed to assess the impact of demographic factors (age and gender) and tooth-specific characteristics on clinical decisions. **Results**: Females, particularly younger, were more likely to choose restorative procedures, while males, especially those over 50, predominantly underwent surgical procedures. Endodontic treatments were most common in males aged 18–30, primarily for maxillary anterior teeth and premolars. Molar extractions, especially in the mandible, were the most frequent surgical procedure. Maxillary teeth, particularly canines and premolars, were more likely to be treated conservatively. **Conclusions**: Economic factors, limited treatment access, and variations in patient preference influenced the observed patterns. Despite a national trend toward increased conservative treatments, disparities persisted based on age, gender, and tooth type. These findings emphasize the need for targeted prevention strategies and equitable access to advanced dental care.

## 1. Introduction

The general health status and health behaviors of individuals are shaped by various factors, both external and individual. While some of these factors, such as age or gender, are unmodifiable, others, like income, education, and lifestyle choices, can be influenced or modified. Individual characteristics, including gender, age, income, and education, play a significant role in determining health behaviors and decisions related to oral health and dental care. Research highlights the impact of these factors on oral health. Poor oral hygiene and less engagement in health maintenance are often linked to male gender, low income, rural residential area, and lower educational attainment [1,2,3,4,5,6,7].

Gender, in particular, stands out as a strong determinant. Males generally exhibit poorer oral health, less consistent hygiene practices, and fewer or less regular dental check-ups and treatment compared to females. These findings underscore the need for gender-specific strategies to enhance oral health and address gender disparities. Beyond hygiene habits, gender also influences emotional well-being and perceptions of oral health. Studies associate poorer self-assessment of oral health and higher dental anxiety levels with males, who also tend to have a greater prevalence of general diseases [5,6,8,9,10].

Dental fear remains a significant barrier to seeking care. Encouragingly, recent trends indicate a reduction in dental anxiety among schoolchildren and young adults benefiting from public dental health programs that emphasize prevention and psychological support. Despite these advancements, dental fear persists at concerning levels, necessitating further refinement of psychological approaches to patient care [11]. Additional determinants of health behaviors include living conditions, sociocultural factors, and access to healthcare services. These factors significantly affect patients’ overall health and their willingness to engage in oral health practices [1,2,12]. The interplay of these external and individual influences highlights the complex nature of medical decision making and the importance of addressing diverse factors to improve health outcomes.

According to the latest report on the oral health of Poles, conducted between 2016 and 2020 in the Greater Poland region, the incidence of tooth decay (dmft/DMFT > 0) showed a concerning increase with age, rising from 21.0% in children aged 3 to 89.0% by the age of 18. Dental calculus was present in one out of four 12-year-olds and nearly half of 18-year-olds. Among adults aged 35–44, the prevalence of dental caries reached a staggering 98.7%, with a dental caries index of 16.5%. Additionally, only 31.5% of individuals in this age group had a healthy periodontium. In older adults aged 65–74, 18.8% were edentulous, and the DMFT index (decayed, missing, and filled teeth) was alarmingly high (24.9). The percentage of individuals with a healthy periodontium was nearly 25%, though edentulism or residual dentition limited precise analysis. The authors of this study highlight some improvements compared to earlier reports on the Polish population, including reductions in the percentage of edentulous individuals, decreased caries intensity, increased retention of natural teeth, reduced treatment needs, and improved periodontal health among adults [1]. However, despite these positive trends, the results remain troubling. The demographic and socioeconomic disparities highlight the need for targeted interventions to bridge gaps in oral healthcare access and outcomes.

Previous studies have shown the influence of global factors on the spectrum of dental procedures performed and thus indicated the need to take a closer look at individual factors depending on patients and their preferences [13,14]. This retrospective analysis aims to explore the impact of factors such as gender and age on the choice of specific dental procedures within restorative dentistry, endodontics, and surgery. Additionally, the study examines the relationship between particular dental services and the treatment of individual tooth groups or the extent of dental procedures required.

## 2. Materials and Methods

### 2.1. Study Design, Setting, and Sample

This retrospective analysis focused on patient records from the University Center of Dentistry and Specialized Medicine (Poznan, Poland). The University of Medical Sciences in Poznan is the leading academic center in central Poland. The analyzed period was between 1 January 2017 and 31 December 2023.

The patient dataset was provided from outpatient departments that maintained operations during the first wave of the COVID-19 pandemic in 2020, specifically the Clinic of Conservative Dentistry and Periodontology, the Clinic of Oral Surgery, and the Central Dental Clinic. All patients from these three clinics, both children and adults, were included. The number of patients was determined by year, divided by gender and age (Table 1).

### 2.2. Data Collection

The medical database of patients was based on the software KS-SOMED v.2024.03.0.07 (Kamsoft, Katowice, Poland). The system generated data on all visits from the examined period, taking into account demographic data (gender, age on the day of the visit) and visit data (date, clinic, performed procedure based on ICD-9, location in oral cavity). Selected procedures in restorative dentistry (i.e., temporary fillings, refunded, or commercial restorations), endodontics (i.e., intervention procedures, root canal fillings), and dental surgery (i.e., extractions) were analyzed in detail.

### 2.3. Data Analysis

The radar plot was used to present the percentages of procedures performed in the specific tooth number. Next, the distributions of these specific procedures were demonstrated depending on the gender and age group of patients, as well as the extent of them. The proportions between the two groups were compared with the Z test for independent proportions.

Also, the odds ratios were calculated for the surgical procedures vs. the conservative procedures (restorative and endodontic procedures) using univariate logistic regression modeling. To explore the relationship between personal factors and the spectrum of dental procedures, the multidimensional correspondence analysis was performed using the two-dimensional visualization based on the scree plot. The smaller the angle between the evaluated points, the stronger the dependence (with the vertex of the angle placed at the origin of the coordinate system).

The significance level was set at alfa = 0.05. All analyses were conducted using Statistica v.13.3 (Statsoft, Cracow, Poland) and PQStat v.1.8.6.122 (PQStat Software, Poznan, Poland).

## 3. Results

The number of patients categorized by gender and age for the years 2017–2023 is presented in Table 1. It is evident that in every period, females outnumbered males across all age groups. The lowest number of patients was recorded in 2020, likely due to the COVID-19 pandemic, while a significant increase in patient numbers was observed in 2022–2023, following Russia’s invasion of Ukraine.

**Figure 1:**
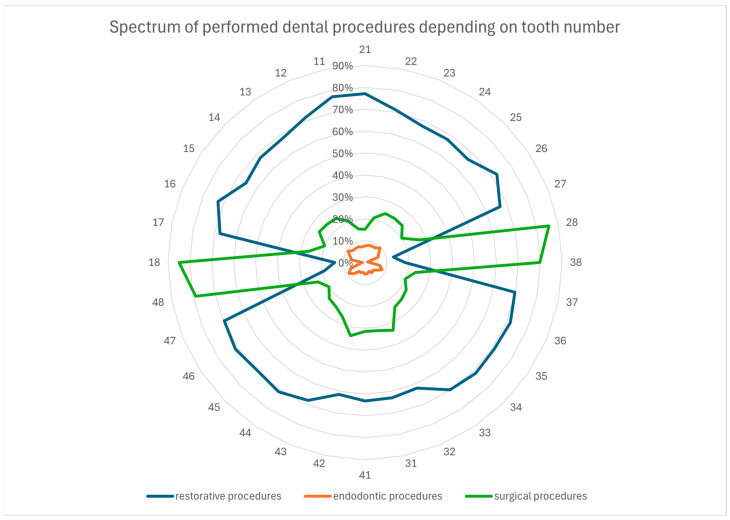
Radar plot for the percentage spectrum of performed dental procedures depending on the tooth number (as outer numbers according to the FDI numbering system).

This study’s analysis focused on the most common procedures performed on permanent teeth: conservative treatments (restorations and root canal fillings) and surgical procedures (extractions). A total of 182,654 patients’ medical records from the University Center of Dentistry and Specialized Medicine (Poznan, Poland) were analyzed. The radar chart (Figure 1) illustrates the percentage distribution of these three treatment procedure groups depending on tooth numbers. The vast majority of procedures involved tooth restorations, except for wisdom teeth, which were most frequently extracted. Endodontic procedures were notably less common. The size of the chart fields highlights a tendency for more frequent conservative procedures in maxillary teeth compared to mandibular teeth.

The comparative analysis of gender’s influence on the spectrum of dental procedures performed on individual teeth is shown in Table 2. Overall, females were more likely than males to opt for tooth restorations, regardless of tooth type. Interestingly, only males more frequently chose conservative treatment for wisdom teeth. Conversely, surgical procedures were generally more common among males, except in the case of wisdom teeth. Additionally, males were more prone to experiencing tooth pain that culminated in endodontic intervention, particularly in the anterior teeth and premolars of the maxilla.

On the other hand, a similar comparison considering the age of patients is provided in Table 3. As age increased, the percentage of teeth treated conservatively decreased, while the percentage of teeth removed increased. This trend was consistent across all teeth, except for wisdom teeth, which showed the opposite pattern compared to other teeth. Regarding endodontic treatment, interventional procedures were most commonly performed in the 18–30 age group. However, the percentage of teeth ultimately treated endodontically was slightly higher in the 18–30 and 31–50 age groups. Again, these procedures were predominantly performed on anterior teeth and maxillary premolars.

The extent of restorations performed and the type of refoundation, depending on tooth number, are summarized in Table 4. Single- and two-surface restorations were the most frequently performed. In the majority of mandibular teeth, single-surface restorations predominated. In contrast, maxillary teeth, particularly anterior and premolars, showed a preference for two-surface restorations. Notably, commercial restorations were more frequently chosen for maxillary premolars with two-surface cavities. Multi-surface restorations were the least common overall, but within this group, more than one-third were commercial, especially in the maxillary anterior teeth.

Surgical extractions were the most frequently performed surgical procedures, as shown in Table 5. These primarily involved molar teeth, with mandibular wisdom teeth being the most commonly extracted.

Based on univariate logistic regression modeling, the odds ratios for surgical procedures compared to conservative procedures were assessed depending on personal factors (Table 6). Maxillary teeth were significantly less likely to be qualified for extractions. Molar teeth had the highest odds of extraction, in contrast to incisors, which had the lowest. Among demographic factors, females were 8.5% less likely to decide on tooth extractions compared to males. On the other hand, regarding age, teeth were most frequently extracted in patients over 51 years of age, with odds for extraction being 6% higher than in the 18–30 age group.

**Figure 2:**
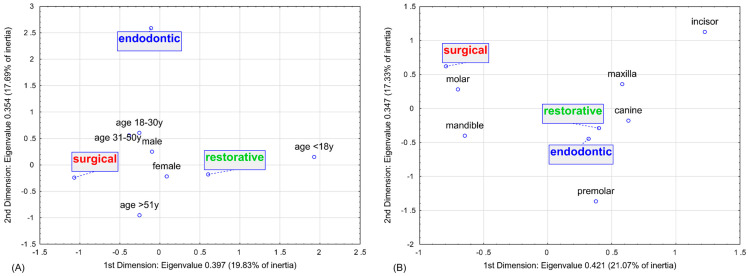
Multidimensional correspondence analysis for impact of (**A**) sociodemographic factors (i.e., age and gender) and (**B**) personal oral factors (i.e., tooth type and site) on the spectrum of dental procedures—2-dimensional plot.

Multidimensional correspondence analyses were conducted to graphically represent the relationship between individual factors and clinical decisions regarding the type of dental procedure. Based on scree plot evaluation, a two-dimensional analysis was selected for both demographic and personal oral factors (Figure 2).

The results revealed that males over 50 years of age were most likely to choose surgical procedures. In contrast, females—especially those under 18 years of age—more frequently opted for restorative procedures. Interestingly, males aged 18–30 years were the group most likely to undergo endodontic treatment.

In terms of tooth type and location, the analysis confirmed that mandibular molars were the most predisposed to extraction. Conversely, maxillary teeth—particularly canines and premolars—were more commonly selected for conservative procedures. Detailed parameters of the analysis are presented in Tables S1 and S2.

## 4. Discussion

A total of 182,654 medical records of patients treated at the University Center of Dentistry and Specialized Medicine in Poznan (Poland) between 2017 and 2023 were analyzed. During this period, two significant global events heavily influenced the number of dental services provided. The first quarter of 2020 saw the largest decline in dental services due to the rapidly spreading COVID-19 pandemic, which exposed the unpreparedness of healthcare systems worldwide. In response to the crisis, our center, following the recommendations of leading medical and dental societies, restricted its services to emergency care only during the early months of the pandemic [15,16,17]. This decision resulted in a drastic reduction in the number of patients admitted during this period [13,18,19,20]. In turn, on 24 February 2022, the war in Ukraine triggered a massive influx of refugees into neighboring Poland, driven by both the country’s geographical proximity and cultural and linguistic similarities. In response, Polish authorities swiftly implemented mechanisms to assist those in need, including access to reimbursed medical services. This policy led to the largest recorded increase in dental services provided, as many refugees sought dental care at our center [21,22,23].

In addition to these global events, a long-term trend in Poland has also impacted the provision of dental services. Each year, an increasing number of dental practices previously offering treatments reimbursed by public funds have been closed or privatized. This shift is driven by factors such as the rising costs of maintaining dental clinics and acquiring materials, as well as the increasing expectations of patients, who demand higher-quality and more personalized services [24]. In this changing landscape, our center has emerged as the largest medical referral center providing specialist dental care reimbursed by the National Health Fund in central Poland. As a result, it has attracted a growing number of patients seeking free treatment, which is often no longer available in their local areas. Consequently, our center has experienced a steady upward trend in the number of procedures performed, reflecting its critical role in meeting the dental care needs of both Polish residents and refugees.

### 4.1. Sociodemographic Factors (i.e., Gender and Age)

Looking closely at the details of the procedures performed over these seven years, the clear dominance of females stands out across the entire study period and in every age group analyzed. The literature frequently highlights females’ more frequent use of medical services, including dental care, which aligns with the study’s findings [3,4,5,6,9]. This phenomenon reflects well-documented gender patterns. Furuta et al. suggest that oral health behaviors are influenced by various factors such as knowledge, attitude, lifestyle, stress, education level, and socioeconomic status, with the first three showing notable differences between genders [7]. Females tend to perceive a stronger connection between oral health and their quality of life, including mood, appearance, and overall well-being. This awareness translates into more frequent check-ups and adherence to recommended treatments, even when financial barriers are reported [3,5,6,7].

These conclusions are supported not only by survey data but also by the widely used DMF index, a key indicator of dental health in epidemiological studies. Among females, the F (filled) component is often dominant, indicating a higher likelihood of undergoing dental treatment for caries, while the M (missing) component is typically smaller, reflecting a lower tendency to remove teeth due to decay [1,4,25,26,27]. In this study’s analysis, females were more likely to choose restorative dental treatments, particularly those under 18 years of age. This aligns with findings from the report “Monitoring the Oral Health Status of the Polish Population in 2016–2020”. According to this report, the conservative treatment indicator—which reflects not only the availability and accessibility of preventive and therapeutic services but also patients’ own concern for their oral health—was higher among females in both the 35–44 and 65–74 age groups [1].

The available literature clearly demonstrates that females tend to have greater knowledge, a more positive attitude, and a generally healthier lifestyle, which translates into better oral hygiene. This is evidenced by more frequent and thorough tooth brushing, interdental flossing, and less tobacco product use [3,5,6,7,28]. Consequently, females tend to have a healthier periodontium, yet paradoxically, they still experience higher rates of tooth decay [4,6,9,25,26,27,28]. Although some studies present a different perspective, they remain a clear minority [5]. Anthropologists attribute this paradox to three primary factors: earlier eruption of teeth in girls, leading to longer exposure to a cariogenic oral environment; easier access to food supplies and frequent snacking during food preparation; and the physiological and behavioral changes associated with pregnancy [9]. However, Lukacs et al. emphasized that anthropologists tend to focus on social issues while downplaying the less-documented impacts of hormonal factors [4].

Conversely, researchers like Ferraro and Vieira reported limited evidence supporting the idea that earlier tooth eruption significantly contributes to caries development [9]. Instead, growing attention has turned to the role of hormones, particularly estrogen, which fluctuates during puberty, menstruation, and pregnancy. These hormonal changes alter the biochemical composition of saliva, leading to a lower flow rate of both unstimulated whole saliva and stimulated parotid saliva in females. This reduction in saliva flow creates a more cariogenic oral environment due to diminished mechanical washing, buffering, and remineralization benefits provided by saliva [6,9,10,28]. Additionally, factors such as diseases, medical procedures, and medications (e.g., hormone replacement therapy and birth control pills) can further affect the composition and flow rate of saliva [10]. Pregnancy, in particular, has a profound impact on females’ bodies and daily behaviors. It can lead to immune suppression, hormonal fluctuations, cravings, and salivary alterations, all of which contribute to a higher risk of caries [9,28,29]. Studies also indicate that males have higher concentrations of IgA immunoglobulin in their saliva, aiding in defense against cariogenic pathogens [30,31]. Moreover, females tend to have a deficient amelogenin gene and lower levels of amelogenin protein, which are critical for forming enamel matrix and may contribute to increased caries susceptibility [32,33].

Salivary composition, which is regulated by hormonal and biochemical factors, also differs between genders and may influence caries risk. Considering these biological differences, personalized treatment approaches could improve oral health outcomes. For a deeper exploration of gender as a biological variable in oral diseases, the recent review by Sangalli et al. provides valuable insights into these mechanisms [34].

There is also the other side of the coin. Males generally use healthcare services less often, including dental care, and when they do, it is typically for acute problems, such as dental pain, rather than for preventive care [5,6]. This pattern is also evident in this study’s analysis. At our center, males were more likely to report with toothaches, often resulting in emergency procedures. Notably, the group of males aged 18–30 was found as the most likely to undergo interventional endodontic procedures. Males also more frequently opted for surgical procedures, which are often performed during pain visits. This tendency may stem from a lack of willingness to pursue conservative treatment or from the extent of tooth damage, where tooth extraction becomes the only viable option. Unfortunately, the study dataset lacks more detailed information on the underlying reasons for these choices.

The tendency to postpone treatment seems to be linked to “traditional masculine behavior”, “masculinity beliefs”, and the association of illness with a loss of masculinity. However, Galdas et al. suggested that occupational and socioeconomic status likely have a greater influence on these behaviors than gender alone, highlighting the need for further investigation [35,36]. Males are also characterized by less knowledge of proper oral hygiene practices. They tend to use harder toothbrushes, brush with excessive force, and are less likely to use recommended fluoride toothpaste or floss interdental spaces. These habits can lead to gingival damage and recession, contributing to the development of root caries [6,37]. This is supported by the observations of Su et al., who found that males are more prone to root caries, noncarious root lesions (such as erosions or abrasions), and root restorations [5].

Age appears to be another important factor influencing the choice of treatment method. As patients age, the percentage of teeth treated conservatively decreases (70.9% vs. 55%), while the number of teeth removed increases (9.7% vs. 34.9%), consistent with other reports [1,26,27,38]. With age, the percentage of third molars removed decreases, although it remains the highest among all tooth groups (65.3–76.3%). Endodontic procedures, however, were very rare across all age groups.

According to the report “Monitoring the Oral Health of the Polish Population in 2016–2020”, the incidence of caries in children in Greater Poland rises from 21% in the group up to 3 years old to 89% by the age of 18. Treatment needs in this age group most often involve single- and two-surface fillings, while the demand for endodontic treatment and tooth extractions peaks at 9.2% in 10-year-olds. During the mixed dentition period, one in three children require tooth extraction. The report highlights the low conservative treatment rate for primary teeth (0.31 in 3- and 10-year-olds), indicating that treatment needs are insufficiently met. This rate increases 2.5 times for permanent teeth by the age of 18 (0.84) [1].

In Poland, among individuals aged 35–44, the prevalence of caries remains at an alarmingly high level of 98.7%. In Greater Poland, unfavorable changes in the conservative treatment index were observed, decreasing from 0.64 in 2010 to 0.56 in 2017. Among older adults aged 65–74, the percentage of edentulous individuals dropped to 18.8%, while the percentage of people with at least 20 preserved teeth increased from 8% to 27%. Despite these improvements, the DMFT index for seniors remains at a very high level of 24.9. However, the conservative treatment index rose to 0.67 nationally and 0.8 in the Greater Poland province [1].

Zilinskaite-Petrauskiene et al. described differences in factors influencing endodontic treatment between elderly and young patients. In older patients, a significantly higher proportion presented with necrotic pulp and required both endodontic and prosthetic treatment. With age, the difficulty of performing endodontic procedures increases due to challenges such as achieving proper access and locating root canal orifices. However, there were no significant differences between the two groups in terms of the number of treatment visits, the technical quality of root fillings, pain sensation, esthetic outcomes, or masticatory function [39].

The most common general reason for tooth extraction remains dental caries. Among younger patients, orthodontic indications are cited more frequently, whereas in older patients, periodontal disease becomes a more prevalent cause. This topic is explored in more detail later [38,40,41,42,43,44].

Socioeconomic factors significantly influence dental patients’ decisions between conservative and surgical procedures. Individuals with higher income and education levels, as well as those residing in urban areas, often have better access to dental care and greater awareness of oral health, leading them to prefer conservative treatments. Conversely, those with lower socioeconomic status may face barriers such as cost and limited access, resulting in delayed care and a higher likelihood of requiring surgical interventions [45]. Occupational factors also play a role in treatment choices. For instance, individuals in demanding jobs or with irregular work schedules may opt for quicker solutions like extractions to minimize time away from work, whereas those in professions that prioritize esthetics may choose conservative procedures to maintain their appearance [46]. Future research should delve deeper into these socioeconomic and occupational influences to develop targeted strategies that improve access to and utilization of conservative dental care, particularly among vulnerable populations.

### 4.2. Personal Oral Factors (i.e., Tooth Group, Site, or Procedure Extent)

The vast majority of dental procedures performed across all dental groups at our center were dental restorations, with the notable exception of wisdom teeth, where extractions were the predominant treatment. In Poland, there has been a positive trend in recent years toward increasing the conservative treatment rate—defined as the ratio of teeth with properly made restorations to the total number of teeth filled and those requiring treatment [1]. This trend is somewhat encouraging, reflecting an improvement in access to and prioritization of restorative care. According to the medical records we analyzed, restorative treatment was most commonly performed on maxillary teeth, particularly on canines and premolars. In the mandible, single-surface restorations were more frequent, while in the maxilla, two-surface restorations were more common, especially in the anterior teeth and premolars.

There are reports indicating that permanent dental caries in children primarily affects molars, often appearing shortly after their eruption. For example, the first molar is affected in children as young as 5 years old, while the second molar tends to show caries in 12-year-olds [1]. Pizzo et al. reported that 44% of current active caries foci were found in the first molars of 6–7-year-olds based on the analysis of 742 subjects [47]. Similarly, Alves et al. found 17.2% of current active caries foci in second molars among 983 examined 12-year-olds [48].

The early onset of caries brings significant consequences in adulthood. Once a tooth is drilled, it often enters what is known as the “death spiral of restoration” or the “tooth cycle of death” [49]. This cycle involves repeated restorations, gradually leading to the loss of more tooth tissue until, eventually, endodontic–prosthetic treatment is required on the dental pillar, or in extreme cases, implant–prosthetic treatment after the tooth has been extracted. The sooner the “first drilling” occurs, the sooner the tooth may ultimately require removal. This process is associated with rising treatment costs. Unfortunately, under the National Health Fund in Poland, endodontic treatment is only covered for anterior teeth (“from canine to canine”), and fixed prosthetics are not reimbursed at all [50,51]. The long waiting times for refunded treatments, combined with the risk of pain or swelling from untreated teeth, add to the difficulty for patients [24]. Those with issues concerning posterior teeth, which often require more advanced or specialized care, are left dependent on commercial treatment if they wish to preserve their teeth. Additionally, the microscopic magnification of dental procedures, especially for molars with multiple roots (3–4) compared to the fewer roots (1–2) of front teeth, has been highlighted [52,53,54,55]. This approach not only increases treatment complexity but also raises the cost of the service. The subsequent need for the prosthetic reconstruction of crowns destroyed by caries adds to the financial burden. Many patients simply cannot afford these treatments [24].

On the other hand, tooth extractions are fully reimbursed, including the surgical removal of impacted teeth. Studies have shown that wealthier individuals tend to have higher treatment rates and higher F components in the DMF (decayed, missing, filled) caries index, while those with lower incomes are more likely to experience a higher M component, indicating a preference for extraction over attempts to preserve teeth. This disparity clearly suggests that many people with limited financial resources opt to extract teeth rather than invest in treatments to save them [1,25,27,56].

According to the study results, endodontic procedures, which are the last resort to preserve one’s own teeth, were significantly less common compared to other dental services. These interventional procedures were most frequently performed in the 18–30-year age group. However, in the case of teeth with root canals that were ultimately filled, the percentage was notably small, with minimal differences across the age groups studied (2.6% vs. 2.6% vs. 2.8% vs. 2.8%). This procedure primarily involved the anterior and premolar teeth of the maxilla, with a slight gender difference (2.8% in males vs. 2.4% in females). The dominance of endodontic procedures in these two groups of teeth contrasts with the findings of other researchers, who typically report molars as the teeth most predisposed to root canal treatment, sometimes alongside premolars [57,58,59,60]. These studies also generally indicated a predominance of endodontic treatment in females [57,59]. Although these studies were conducted on relatively small research groups compared to this study’s extensive database, it is believed that economic factors may have influenced the notably low percentage of endodontic procedures in this analysis. Unfortunately, due to the retrospective nature, the study lacks data on factors such as the economic status of patients, which could have further supported this hypothesis.

When surgical procedures are taken into account, the highest percentage of teeth removed in this study concerns molars, particularly lower molars in adults, which aligns with numerous other studies [25,26,27,40,41,42,61]. Surgical extractions were performed more frequently (79.4% vs. 20.6%) across all tooth groups, except lower teeth. This can be explained by the nature of our facility as a reference center, where more complex cases requiring specialized care are referred. In their reviews, Gotfredsen et al. and Elias et al. emphasize that the loss of anterior teeth has the greatest impact on esthetic and psychological issues, while the preservation of premolars tends to have a greater impact on patient satisfaction than the preservation of molars [62,63]. In contrast, the loss of posterior teeth does not appear to have significant subjective effects for patients. Older adults with shortened dental arches, meaning they have remaining anterior and premolar teeth, maintaining masticatory function, occlusal support, and dental arch stability [25].

In numerous studies, dental caries is identified as the primary cause of tooth extraction [40,41,42,43,44]. It is possible that the high percentage of molar extractions in adulthood is related to the early appearance of carious lesions shortly after the eruption of these teeth and the early onset of the so-called “death spiral” of tooth restoration. Multi-rooted teeth, such as molars, may seem more difficult to extract compared to single- or two-rooted teeth, so the specialized nature of our center may also be a contributing factor. McCaul et al. indicated molars as the most frequently extracted teeth in adults up to the age of 50. Before the age of 21, it was primarily premolars, especially the first upper premolars, and after the age of 50, more anterior than posterior teeth were extracted [41].

Dental caries was identified as the main cause of tooth extraction for nearly all tooth groups. The exceptions were the lower incisors, which were extracted for prosthetic reasons (other than caries or periodontitis), and premolars in people under 21, where the primary reason was orthodontic. Other researchers have also noted similar reasons for tooth extraction in various tooth groups [38,40,43]. The reason for the loss of front teeth at a later age has been explained by esthetic factors, easier access to conservative and endodontic treatments, and their greater resistance to caries, but less resistance to periodontal diseases, which tend to manifest later in life. This study dates back to 1999, and at that time, the authors observed a rise in interest in orthodontic treatments, leading to an increase in the number of premolar extractions compared to 1984 in younger people. There is currently an ongoing debate between the extraction vs. non-extraction of premolars in orthodontic treatments. At present, there is no conclusive evidence supporting the superiority of either method [64]. However, contemporary studies indicate a decreasing trend in the tendency to extract premolars before orthodontic treatment [65,66,67]. For example, Dardengo et al. analyzed this topic over a broad period from 1980 to 2011 and observed a reduction of about 20% in the frequency of cases requiring tooth extraction over the last 32 years [65]. Additionally, Fleming et al., based on surveys, noted a significant reduction—approximately 95%—in the tendency to prescribe orthodontic extractions over the past 5–10 years among British Orthodontic Society members [67]. In this study, the percentage of premolars removed was significantly lower in the <12, 18–30, and 31–50 age groups compared to the >51 group. This suggests a slight tendency to extract premolars for orthodontic reasons in the study population.

As previously mentioned, the largest percentage of services was tooth restorations, with the exception of wisdom teeth, which were most frequently removed (75.4–88.3%). These teeth were also the most commonly extracted across all age groups studied—removals outnumbered those of individual first molars by more than double. This result is not surprising. Dental caries is commonly cited as the primary cause for the extraction of these teeth [40,41,43,60]. Their position in the oral cavity is undoubtedly a contributing factor: located at the back of the dental arch, often tilted toward the cheek and frequently only partially erupted, they are difficult for patients to clean effectively. This makes them prone to the accumulation of food debris, which serves as a breeding ground for cariogenic bacteria. Conservative treatment is also challenging for dentists for similar reasons. Inadequate cleaning of these teeth promotes the stagnation of calculus, leading to periodontal disease, which is another common reason for their removal [68,69,70,71].

Furthermore, third molars often experience complete or partial impaction due to insufficient space in the dental arches. This can result in conditions such as pericoronitis, mandibular angle fractures, cysts, tumors, or damage to the adjacent molars, which may prompt clinicians to recommend prophylactic removal, even though there is currently no evidence to support the routine extraction of asymptomatic impacted teeth in adults. It is worth noting that impaction is seven times more common in the lower third molars. The tendency for impaction is attributed to the progressive agenesis of these teeth, due to their limited functionality in modern chewing physiology [68,70,71].

Adeyemo et al., based on an analysis of 1763 cases of third molar extractions, reported that caries was the primary cause for removal in 63.2%, recurrent pericoronitis in 26.3%, and periodontitis in 9.2%, with only 0.6% of extractions being prophylactic. The study emphasized that patients who had third molars removed due to caries were statistically younger than those who had them extracted due to periodontitis, but older than those with extractions due to pericoronitis [68]. Moreover, Ventä et al., based on an analysis of 6082 panoramic radiographs, identified third molars (both maxillary and mandibular) as the most commonly missing teeth up to the age of 80. They reported agenesis of all four wisdom teeth at a rate of just 3.4%, with at least one wisdom tooth missing in 22.6% of the cases [61]. Their findings were consistent with the results of a 1999 study, which examined a massive database of 100 million dental procedures among 7.5 million patients in the United States [72].

However, Baqain et al. indicated that upper wisdom teeth were extracted the least frequently [40]. It is important to note that this study was conducted at an oral surgery teaching clinic, where many extractions of third molars may have been excluded from the analysis due to the need for greater operator experience, which could have influenced the final outcome. When reviewing the literature, it is crucial to consider whether third molars were excluded from the analysis entirely.

### 4.3. Study Strengths, Limitations, and Future Directions

This study boasts several strengths, including the analysis of an extensive dataset comprising 182,654 medical records collected over a 7-year period (2017–2023). This timeframe covers critical global events, such as the COVID-19 pandemic and the outbreak of the Russia–Ukraine war, allowing for a comprehensive evaluation of long-term changes in dental healthcare.

However, the study also has its limitations. Key socioeconomic factors, such as patients’ wealth, place of residence, and education level, were not available in the dataset, which restricted a more in-depth analysis of these important determinants. As the largest dental facility in central Poland, our center provides care to a diverse patient population, encompassing individuals from urban and rural areas as well as various socioeconomic backgrounds. However, this single-center setting may limit the generalizability of the study results. Additionally, the study did not include all clinics operating within the University Center of Dentistry and Specialized Medicine because several departments interrupted their services during the first wave of COVID-19.

Future research should aim to address these limitations by incorporating a more comprehensive dataset that includes socioeconomic factors such as income, education level, and place of residence. A multicenter approach involving other dental facilities across different regions would enhance the generalizability of the findings. Further studies should delve deeper into the socioeconomic, educational, and occupational influences to develop targeted strategies that improve access to and utilization of conservative dental care, particularly among vulnerable populations.

## 5. Conclusions

Consistent with the existing literature, this study’s findings confirm that females and younger individuals are more likely to seek preventive and conservative services, while males are more likely to undergo interventional endodontic and surgical procedures. The frequency of surgical treatments increased with age, whereas conservative procedures declined. Restorative dentistry services were the most frequently performed procedures, particularly single- and two-surface restorations in maxillary teeth. Among surgical procedures, surgical extractions were more commonly performed than simple extractions, most frequently involving mandibular molars.

These results underscore the need for targeted interventions to improve oral health outcomes, particularly among males and older individuals, who are less likely to engage in preventive care. Strengthening efforts to promote conservative treatment and expanding access to guaranteed dental services are essential for reducing disparities and ensuring equitable oral healthcare for all socioeconomic groups.

## Figures and Tables

**Table 1:** The number of patients from selected outpatient departments in the University Center of Dentistry and Specialized Medicine (Poznan, Poland) from 1 January 2017 to 31 December 2023.

	0–18		19–30		31–50		>51	
	F	M	F	M	F	M	F	M
**2017**	792	667	4800	3773	4716	3932	5354	3741
**2018**	715	629	4584	3499	4367	3711	4982	3488
**2019**	661	630	4468	3503	4305	3929	5044	3963
**2020**	568	461	3064	2175	3255	2935	3133	2216
**2021**	772	614	4193	2937	4293	3577	3809	3141
**2022**	980	723	4631	3023	5124	4321	4579	3551
**2023**	1138	1024	4563	3111	5764	4610	4861	3750

Legend: F, females; M, males.

**Table 2:** Percentage distribution of performed dental procedures depending on the tooth number and gender.

Tooth	*n*		Filled Permanent Teeth			Temporary Filling			Endodontic Intervention Procedure			Root Canal Filling			Tooth Extraction		
	F	M	F	M	*p*-Value	F	M	*p*-Value	F	M	*p*-Value	F	M	*p*-Value	F	M	*p*-Value
**11**	3052	3064	73.1	67.9	<0.001 *	6.3	7.2	0.176	2.5	3.1	0.179	4.0	4.9	0.100	14.2	17.0	0.003 *
**12**	2462	2316	68.2	61.1	<0.001 *	6.5	7.3	0.301	2.9	3.6	0.199	4.3	4.9	0.357	18.1	23.2	<0.001 *
**13**	2221	2215	65.3	58.8	<0.001 *	5.8	6.3	0.525	2.8	4.4	0.005 *	3.7	4.5	0.205	22.3	26.0	0.004 *
**14**	3193	2904	62.1	56.5	<0.001 *	7.7	8.6	0.216	4.2	4.3	0.896	3.8	3.2	0.230	22.1	27.4	<0.001 *
**15**	3322	2731	56.6	53.2	0.009 *	10.9	9.9	0.221	5.4	4.9	0.415	4.1	4.1	0.948	23.0	27.9	<0.001 *
**16**	5505	4498	61.7	59.4	0.021 *	12.3	11.8	0.464	4.7	5.2	0.270	2.7	2.1	0.061	18.7	21.5	<0.001 *
**17**	3456	2914	59.6	55.2	<0.001 *	10.5	9.3	0.121	4.8	4.4	0.485	1.6	1.7	0.831	23.5	29.4	<0.001 *
**18**	2837	1953	10.3	14.4	<0.001 *	1.7	2.0	0.514	0.6	1.8	<0.001 *	0.1	0.1	0.642	87.3	81.6	<0.001 *
**21**	3031	3077	71.3	68.2	0.009 *	7.0	7.9	0.197	3.1	2.7	0.392	4.5	5.0	0.391	14.2	16.2	0.032 *
**22**	2495	2402	65.9	60.6	<0.001 *	8.0	7.8	0.836	4.0	3.9	0.915	3.8	4.5	0.247	18.4	23.3	<0.001 *
**23**	2244	2330	63.9	56.7	<0.001 *	7.0	7.8	0.329	3.7	4.5	0.198	3.7	4.3	0.338	21.7	26.7	<0.001 *
**24**	2900	2794	62.4	55.5	<0.001 *	8.4	8.8	0.491	4.8	4.9	0.909	3.0	3.5	0.322	21.3	27.3	<0.001 *
**25**	3100	2743	57.7	53.0	<0.001 *	11.3	10.6	0.417	4.8	5.1	0.640	4.2	5.1	0.116	22.0	26.2	<0.001 *
**26**	5135	4337	61.5	58.2	0.001 *	12.9	12.0	0.197	4.8	5.2	0.399	2.2	2.8	0.071	18.5	21.9	<0.001 *
**27**	3477	2918	59.2	54.3	<0.001 *	10.2	9.3	0.244	4.4	4.7	0.607	1.9	1.7	0.615	24.3	30.0	<0.001 *
**28**	2866	2009	8.9	14.3	<0.001 *	1.7	2.6	0.038 *	1.1	1.1	0.889	0.0	0.1	0.329	88.3	81.8	<0.001 *
**31**	1208	1070	61.1	57.9	0.131	3.3	3.6	0.782	2.4	2.5	0.985	2.9	2.6	0.758	30.3	33.4	0.123
**32**	1208	1102	60.9	55.5	0.010 *	3.1	4.5	0.098	1.8	2.7	0.186	1.9	2.4	0.493	32.3	34.8	0.220
**33**	1636	1432	67.9	62.1	<0.001 *	4.0	5.5	0.061	2.5	3.1	0.369	2.3	3.5	0.060	23.3	25.8	0.118
**34**	2368	1924	69.8	63.1	<0.001 *	5.3	3.9	0.037 *	2.7	3.1	0.492	2.3	1.7	0.202	19.9	28.2	<0.001 *
**35**	2923	2399	67.0	60.6	<0.001 *	7.0	6.8	0.817	3.3	4.4	0.044 *	2.4	2.8	0.407	20.3	25.4	<0.001 *
**36**	5871	4993	60.3	55.8	<0.001 *	13.8	13.2	0.377	5.7	5.8	0.856	2.7	2.8	0.796	17.6	22.4	<0.001 *
**37**	4415	3785	61.0	56.1	<0.001 *	11.6	10.4	0.090	5.1	5.4	0.577	1.4	1.7	0.312	20.9	26.4	<0.001 *
**38**	3902	2773	13.6	17.5	<0.001 *	2.9	2.8	0.867	1.7	2.4	0.054	0.0	0.2	0.018 *	81.8	77.1	<0.001 *
**41**	1187	1085	60.5	58.3	0.306	3.6	3.9	0.791	2.1	2.8	0.345	2.4	3.3	0.244	31.3	31.7	0.873
**42**	1140	1080	59.5	55.6	0.069	3.9	3.6	0.795	2.0	1.8	0.850	2.2	3.1	0.234	32.4	35.8	0.100
**43**	1429	1431	66.1	61.2	0.007 *	4.3	4.5	0.865	2.1	2.4	0.678	2.3	3.1	0.228	25.1	28.7	0.033 *
**44**	2297	1908	69.4	62.9	<0.001 *	5.0	4.1	0.189	2.7	3.2	0.387	2.1	1.6	0.282	20.8	28.1	<0.001 *
**45**	2992	2516	66.5	59.2	<0.001 *	7.3	5.6	0.013 *	4.1	4.5	0.507	2.8	3.0	0.718	19.3	27.7	<0.001 *
**46**	5996	5282	59.0	55.4	<0.001 *	14.1	13.8	0.666	5.5	6.4	0.048 *	3.0	2.8	0.565	18.4	21.6	<0.001 *
**47**	4187	3884	61.4	55.6	<0.001 *	11.8	10.1	0.016 *	4.7	5.5	0.113	1.6	2.5	0.005 *	20.5	26.3	<0.001 *
**48**	3876	2854	14.4	17.7	<0.001 *	2.3	4.2	<0.001 *	1.6	2.6	0.005 *	0.1	0.1	0.696	81.6	75.4	<0.001 *
	97,931	84,723	56.0	53.5	<0.001 *	8.4	8.3	0.446	3.8	4.2	<0.001 *	2.4	2.8	<0.001 *	29.4	31.2	<0.001 *

Legend: F, females; M, males; *, significant differences in Z test for independent proportions.

**Table 3:** Percentage distribution of performed dental procedures depending on the tooth number and age.

Tooth	*n*				Filled Permanent Teeth				Temporary Filling				Intervention Procedure				Root Canal Filling				Tooth Extraction			
	<18	18–30	31–50	>51	<18	18–30	31–50	>51	<18	18–30	31–50	>51	<18	18–30	31–50	>51	<18	18–30	31–50	>51	<18	18–30	31–50	>51
**11**	1167	915	1497	2537	77.8	72.6	71.8	65.6	9.3	11.4	6.1	4.3	3.4	5.9	2.9	1.3	7.9	4.9	4.1	2.9	1.6	5.2	15.2	26.0
**12**	510	758	1347	2163	79.6	68.9	64.1	60.2	8.8	11.2	7.6	4.4	3.5	5.5	3.8	2.0	5.3	6.1	4.8	3.7	2.7	8.3	19.7	29.6
**13**	139	384	1199	2714	87.1	67.7	65.7	58.4	2.9	10.2	5.3	6.0	2.2	6.8	4.0	3.1	2.9	5.2	3.8	4.1	5.0	10.2	21.2	28.4
**14**	620	1168	2116	2193	73.1	62.3	58.4	55.1	9.5	12.3	7.5	6.2	3.1	7.0	5.1	2.3	2.4	4.2	3.9	3.1	11.9	14.1	25.2	33.2
**15**	534	1630	2229	1660	70.4	55.7	54.0	50.9	12.4	15.4	9.6	6.0	5.1	7.7	5.2	2.8	3.6	5.8	4.2	2.4	8.6	15.5	26.9	38.0
**16**	3047	2249	2881	1826	72.2	63.1	53.0	50.9	15.4	13.2	11.1	6.6	4.4	6.4	5.7	2.7	2.3	2.9	2.7	1.6	5.8	14.4	27.6	38.2
**17**	853	1567	2286	1664	81.0	70.6	51.4	41.7	13.5	9.6	10.7	7.5	1.4	5.6	5.8	3.7	0.6	1.0	2.6	1.5	3.5	13.2	29.4	45.7
**18**	119	2133	1888	650	5.0	7.3	14.0	22.9	0.0	0.8	2.6	3.2	1.7	0.9	1.4	0.9	0.0	0.0	0.3	0.0	93.3	91.0	81.7	72.9
**21**	1266	875	1575	2392	74.7	74.4	70.4	65.0	11.6	11.9	6.5	4.3	3.9	4.2	3.1	1.7	8.2	5.3	4.3	3.1	1.6	4.2	15.7	26.0
**22**	621	655	1405	2216	76.3	67.8	60.7	60.0	11.4	11.6	8.5	5.4	3.7	5.5	5.7	2.4	5.0	6.1	4.3	3.1	3.5	9.0	20.8	29.1
**23**	127	390	1292	2765	78.7	71.0	59.7	58.1	8.7	7.9	10.0	6.0	2.4	5.9	5.2	3.4	3.9	3.8	4.0	4.1	6.3	11.3	21.1	28.4
**24**	543	1201	2107	1843	71.6	62.4	56.5	56.0	9.8	11.2	9.8	5.3	3.1	8.5	5.2	2.6	2.6	4.4	3.5	2.4	12.9	13.6	25.0	33.7
**25**	528	1567	2184	1564	67.2	56.4	52.8	54.5	13.4	15.3	11.5	5.1	5.7	7.1	4.8	2.6	4.2	6.8	4.6	2.6	9.5	14.4	26.3	35.1
**26**	2712	2263	2791	1706	72.3	63.3	53.2	47.0	16.1	12.8	12.3	6.7	3.5	7.2	5.9	3.0	2.0	3.4	2.8	1.6	6.1	13.4	25.8	41.6
**27**	884	1579	2272	1660	81.6	68.6	49.3	43.3	13.2	10.6	10.2	6.5	1.5	5.0	6.3	3.3	0.0	1.6	3.1	1.4	3.7	14.2	31.1	45.5
**28**	128	2226	1902	619	4.7	7.2	13.6	18.7	0.0	1.3	2.8	3.1	0.0	0.6	1.6	1.8	0.0	0.0	0.1	0.2	95.3	90.8	81.9	76.3
**31**	160	118	408	1592	75.6	63.6	57.4	58.2	6.3	12.7	5.4	2.0	3.8	8.5	2.2	1.9	6.3	9.3	4.4	1.5	8.1	5.9	30.6	36.3
**32**	82	89	344	1795	73.2	66.3	54.9	57.9	7.3	11.2	5.8	2.8	3.7	7.9	3.2	1.7	4.9	3.4	2.6	1.8	11.0	11.2	33.4	35.7
**33**	39	97	489	2443	87.2	80.4	68.9	63.5	7.7	9.3	5.1	4.4	0.0	3.1	2.2	2.9	0.0	1.0	2.2	3.1	5.1	6.2	21.5	26.2
**34**	157	338	1143	2654	78.3	71.0	70.8	63.9	5.1	10.1	4.9	3.9	1.3	5.3	3.3	2.4	1.9	1.2	1.6	2.3	13.4	12.4	19.4	27.5
**35**	306	898	1698	2420	76.5	64.3	64.2	62.4	5.9	13.7	6.5	4.9	4.6	6.6	4.0	2.5	2.9	3.2	2.8	2.1	10.1	12.2	22.5	28.0
**36**	3599	2813	3047	1405	69.7	57.3	49.6	49.2	16.1	13.8	12.8	8.2	4.3	7.8	6.1	4.3	1.7	4.1	3.1	2.1	8.2	17.0	28.4	36.2
**37**	1477	2172	2758	1793	77.0	64.8	52.3	46.2	15.2	11.6	10.8	7.5	2.3	6.0	6.9	4.1	0.7	1.2	2.4	1.3	4.8	16.4	27.6	40.9
**38**	355	2739	2519	1062	2.3	10.6	17.3	26.4	0.6	1.4	3.8	5.3	0.3	1.3	2.8	2.6	0.0	0.0	0.0	0.5	96.9	86.7	76.1	65.3
**41**	172	138	410	1552	75.6	67.4	59.0	57.1	7.0	12.3	5.1	2.3	2.9	9.4	3.7	1.4	6.4	7.2	4.1	1.7	8.1	3.6	28.0	37.5
**42**	96	110	336	1678	84.4	79.1	56.5	54.9	5.2	7.3	3.3	3.6	0.0	1.8	2.1	2.0	3.1	6.4	1.8	2.6	7.3	5.5	36.3	37.0
**43**	47	114	475	2224	80.9	85.1	62.1	62.5	10.6	4.4	5.9	4.0	0.0	0.9	2.7	2.3	0.0	4.4	3.2	2.6	8.5	5.3	26.1	28.6
**44**	159	352	1129	2565	76.7	75.0	68.0	64.0	3.8	6.0	5.4	4.1	0.6	5.7	4.0	2.3	0.6	1.4	2.0	1.9	18.2	11.9	20.5	27.7
**45**	368	929	1884	2327	72.8	64.6	63.1	61.2	8.7	11.1	6.7	4.3	4.9	6.1	5.2	2.7	3.8	3.4	3.5	2.0	9.8	14.7	21.5	30.0
**46**	3587	3003	3137	1551	69.6	55.4	49.0	49.5	16.9	15.1	12.2	8.6	3.8	8.7	6.8	3.8	2.1	4.2	3.1	2.0	7.6	16.7	29.0	36.2
**47**	1429	2132	2714	1796	76.3	66.5	51.3	46.3	15.9	10.8	11.1	7.1	2.7	5.8	6.7	3.6	1.3	2.2	2.4	1.9	3.8	14.8	28.5	41.0
**48**	379	2748	2540	1063	2.9	10.2	18.8	27.8	0.3	2.3	3.9	4.5	0.0	1.2	3.2	2.0	0.0	0.0	0.1	0.3	96.8	86.4	73.9	65.5
	26,210	40,350	56,002	60,092	70.9	50.3	50.4	55.0	13.4	9.7	8.4	5.1	3.4	5.3	4.8	2.6	2.6	2.8	2.8	2.4	9.7	31.9	33.6	34.9

**Table 4:** Percentage distribution of performed restorative procedures depending on the tooth number, the extent of the procedure, and the kind of refoundation.

Tooth	*n*	Single-Surface				Two-Surface				Multi-Surface			
		Commercial	Refunded	*p*-Value	Sum	Commercial	Refunded	*p*-Value	Sum	Commercial	Refunded	*p*-Value	Sum
**11**	4311	5.3	28.0	<0.001 *	33.2	6.2	41.6	<0.001 *	47.9	5.2	13.7	<0.001 *	18.9
**12**	3094	5.1	35.4	<0.001 *	40.5	6.2	41.6	<0.001 *	47.8	4.5	7.2	<0.001 *	11.7
**13**	2754	6.0	41.1	<0.001 *	47.1	6.7	38.6	<0.001 *	45.4	3.1	4.4	0.014 *	7.5
**14**	3625	5.5	32.9	<0.001 *	38.3	13.0	37.1	<0.001 *	50.1	3.3	8.3	<0.001 *	11.6
**15**	3333	5.9	25.0	<0.001 *	30.9	14.4	41.9	<0.001 *	56.3	3.7	9.1	<0.001 *	12.8
**16**	6073	5.5	40.6	<0.001 *	46.1	6.5	34.6	<0.001 *	41.1	5.8	7.0	0.008 *	12.8
**17**	3668	6.8	38.3	<0.001 *	45.1	7.4	34.3	<0.001 *	41.6	6.2	7.1	0.134	13.3
**18**	575	8.5	33.0	<0.001 *	41.6	6.3	41.9	<0.001 *	48.2	3.5	6.8	0.016 *	10.3
**21**	4260	5.1	28.5	<0.001 *	33.6	6.1	41.1	<0.001 *	47.2	4.8	14.4	<0.001 *	19.2
**22**	3100	5.8	35.3	<0.001 *	41.1	6.6	40.9	<0.001 *	47.5	4.6	6.8	<0.001 *	11.4
**23**	2755	7.2	41.9	<0.001 *	49.1	6.1	37.1	<0.001 *	43.2	3.1	4.6	0.005 *	7.7
**24**	3361	6.0	30.9	<0.001 *	37.0	13.0	39.4	<0.001 *	52.3	2.6	8.1	<0.001 *	10.7
**25**	3245	5.4	24.6	<0.001 *	30.0	14.9	43.2	<0.001 *	58.2	3.5	8.3	<0.001 *	11.8
**26**	5681	4.9	39.0	<0.001 *	44.0	7.4	34.9	<0.001 *	42.3	6.7	7.0	0.551	13.7
**27**	3643	6.5	38.0	<0.001 *	44.4	7.9	33.8	<0.001 *	41.7	6.6	7.2	0.335	13.9
**28**	541	6.8	37.9	<0.001 *	44.7	8.5	35.1	<0.001 *	43.6	3.1	8.5	<0.001 *	11.6
**31**	1357	6.9	55.8	<0.001 *	62.7	2.1	27.6	<0.001 *	29.8	1.2	6.3	<0.001 *	7.5
**32**	1348	5.7	54.6	<0.001 *	60.3	3.2	31.8	<0.001 *	34.9	1.1	3.6	<0.001 *	4.7
**33**	2000	7.4	53.2	<0.001 *	60.6	3.6	30.9	<0.001 *	34.4	1.5	3.6	<0.001 *	5.1
**34**	2868	9.1	50.7	<0.001 *	59.8	6.6	27.1	<0.001 *	33.8	1.6	4.9	<0.001 *	6.5
**35**	3412	8.4	38.7	<0.001 *	47.1	11.0	33.1	<0.001 *	44.1	3.0	5.7	<0.001 *	8.7
**36**	6325	6.3	44.6	<0.001 *	50.9	6.6	30.2	<0.001 *	36.8	5.0	7.3	<0.001 *	12.3
**37**	4816	7.4	41.1	<0.001 *	48.5	8.9	30.9	<0.001 *	39.8	4.7	7.0	<0.001 *	11.7
**38**	1015	8.0	36.7	<0.001 *	44.7	11.4	32.8	<0.001 *	44.2	4.2	6.8	0.013 *	11.0
**41**	1351	6.4	56.6	<0.001 *	63.0	2.7	27.6	<0.001 *	30.3	1.0	5.8	<0.001 *	6.7
**42**	1279	5.9	51.1	<0.001 *	57.0	2.7	33.2	<0.001 *	35.9	1.3	5.8	<0.001 *	7.1
**43**	1821	7.2	51.8	<0.001 *	59.0	4.2	31.3	<0.001 *	35.5	1.5	4.0	<0.001 *	5.5
**44**	2796	9.1	50.2	<0.001 *	59.3	6.3	27.2	<0.001 *	33.5	2.0	5.2	<0.001 *	7.2
**45**	3479	8.5	36.9	<0.001 *	45.5	11.0	33.7	<0.001 *	44.7	3.0	6.9	<0.001 *	9.9
**46**	6463	5.9	44.9	<0.001 *	50.8	6.0	30.9	<0.001 *	36.9	5.0	7.3	<0.001 *	12.3
**47**	4730	6.9	38.7	<0.001 *	45.6	10.1	32.6	<0.001 *	42.7	5.3	6.4	0.025 *	11.8
**48**	1064	8.6	34.7	<0.001 *	43.3	10.8	33.6	<0.001 *	44.4	3.9	8.5	<0.001 *	12.3
	100,143	6.4	39.2	<0.001 *	45.7	8.0	34.9	<0.001 *	42.8	4.2	7.3	<0.001 *	11.5

Legend: *, significant differences in Z test for independent proportions.

**Table 5:** Percentage distribution of performed surgical procedures depending on the tooth number and the extent of the procedure.

Tooth	*n*	Simple Extraction	Surgical Extraction	*p*-Value
**11**	953	37.0	63.0	<0.001 *
**12**	983	31.6	68.4	<0.001 *
**13**	1072	27.1	72.9	<0.001 *
**14**	1500	23.8	76.2	<0.001 *
**15**	1527	25.3	74.7	<0.001 *
**16**	1993	19.6	80.4	<0.001 *
**17**	1670	22.9	77.1	<0.001 *
**18**	4069	14.1	85.9	<0.001 *
**21**	928	36.4	63.6	<0.001 *
**22**	1017	34.2	65.8	<0.001 *
**23**	1109	30.0	70.0	<0.001 *
**24**	1380	23.5	76.5	<0.001 *
**25**	1399	25.5	74.5	<0.001 *
**26**	1901	18.3	81.7	<0.001 *
**27**	1720	21.1	78.9	<0.001 *
**28**	4174	12.7	87.3	<0.001 *
**31**	723	50.1	49.9	0.981
**32**	774	46.8	53.2	0.014 *
**33**	752	40.6	59.4	<0.001 *
**34**	1014	30.5	69.5	<0.001 *
**35**	1201	29.6	70.4	<0.001 *
**36**	2148	16.6	83.4	<0.001 *
**37**	1922	21.4	78.6	<0.001 *
**38**	5327	5.0	95.0	<0.001 *
**41**	716	52.9	47.1	0.032 *
**42**	756	44.8	55.2	<0.001 *
**43**	769	36.5	63.5	<0.001 *
**44**	1014	29.3	70.7	<0.001 *
**45**	1276	27.5	72.5	<0.001 *
**46**	2244	15.5	84.5	<0.001 *
**47**	1880	18.3	81.7	<0.001 *
**48**	5314	5.5	94.5	<0.001 *
	55,225	20.6	79.4	<0.001 *

Legend: *, significant differences in Z test for independent proportions.

**Table 6:** Odds ratios for surgical procedures vs. conservative procedures (restorative and endodontic procedures) depending on site, tooth type, gender, and age.

	Reference	Beta	SE	*p*-Value	OR	−95%CI	+95%CI
**site: maxilla**	mandible	−0.185	0.010	<0.001 *	0.831	0.814	0.848
**tooth: incisor**	molar	−0.796	0.015	<0.001 *	0.451	0.438	0.465
**tooth: canine**	molar	−0.648	0.020	<0.001 *	0.523	0.503	0.544
**tooth: premolar**	molar	−0.664	0.013	<0.001 *	0.515	0.501	0.528
**gender: female**	male	−0.088	0.010	<0.001 *	0.915	0.897	0.934
**age: <18**	18–30	−1.468	0.024	<0.001 *	0.230	0.220	0.241
**age: 31–50**	18–30	0.059	0.014	<0.001 *	1.061	1.032	1.091
**age: >51**	18–30	0.060	0.014	<0.001 *	1.062	1.034	1.092

* significant ratio for univariate logistic regression modeling.

## Data Availability

The original contributions presented in the study are included in the article; further enquiries can be directed to the corresponding authors.

## Supplementary Materials

The following supporting information can be downloaded at https://www.mdpi.com/article/10.3390/jcm14051508/s1: Table S1: Detailed parameters of determined points in multidimensional correspondence analysis for the impact of sociodemographic factors (i.e., age and gender) on the spectrum of dental procedures; Table S2: Detailed parameters of determined points in multidimensional correspondence analysis for the impact of personal oral factors (i.e., tooth type and site) on the spectrum of dental procedures.
