# Supplementary material for: Factors Influencing the Choice of Conservative and Surgical Procedures in Dental Patients from Poland: A Single-Center Retrospective Analysis
Source: J Clin Med. 2025 Feb 24;14(5):1508. doi: 10.3390/jcm14051508 (PMC11900581; doi:10.3390/jcm14051508)
Supplement: Supplementary file 1 [file jcm-14-01508-s001.zip › jcm-3475521-supplementary.pdf]

Supplementary materials

Łaganowski K, Ortarzewska M, Cieślik K, Jankowski J, Nijakowski K.

Factors Influencing the Choice of Conservative and Surgical Procedures in Dental Patients from Poland: A Single-Center Retrospective Analysis

Table S1. Detailed parameters of determined points in multidimensional correspondence analysis for the impact of sociodemographic factors (i.e. age and gender) on the spectrum of dental procedures.

| | x | y | quality | relative inertia | x inertia | x cos^2 | y inertia | y cos^2 |
|---|---|---|---|---|---|---|---|---|
| age <18y | 1.926 | 0.152 | 0.586 | 0.144 | 0.423 | 0.582 | 0.003 | 0.004 |
| age 18-30y | -0.257 | 0.607 | 0.121 | 0.130 | 0.012 | 0.018 | 0.076 | 0.103 |
| age 31-50y | -0.386 | 0.559 | 0.204 | 0.116 | 0.038 | 0.066 | 0.090 | 0.138 |
| age >51y | -0.255 | -0.951 | 0.501 | 0.110 | 0.019 | 0.034 | 0.290 | 0.467 |
| males | -0.099 | 0.251 | 0.063 | 0.089 | 0.004 | 0.008 | 0.028 | 0.055 |
| females | 0.085 | -0.217 | 0.063 | 0.077 | 0.003 | 0.008 | 0.024 | 0.055 |
| surgical procedures | -1.069 | -0.239 | 0.591 | 0.112 | 0.317 | 0.563 | 0.018 | 0.028 |
| endodontic procedures | -0.111 | 2.589 | 0.520 | 0.155 | 0.001 | 0.001 | 0.454 | 0.519 |
| restorative procedures | 0.603 | -0.179 | 0.589 | 0.067 | 0.183 | 0.542 | 0.018 | 0.048 |

Supplementary materials
Łaganowski K, Ortarzewska M, Cieślik K, Jankowski J, Nijakowski K.
Factors Influencing the Choice of Conservative and Surgical Procedures in Dental Patients from Poland: A Single-Center Retrospective Analysis

Table S2. Detailed parameters of determined points in multidimensional correspondence analysis for the impact of personal oral factors (i.e. tooth type and site) on the spectrum of dental procedures.

| | x | y | quality | relative inertia | x inertia | x cos^2 | y inertia | y cos^2 |
|---|---|---|---|---|---|---|---|---|
| molar | -0.701 | 0.281 | 0.584 | 0.082 | 0.197 | 0.503 | 0.039 | 0.081 |
| premolar | 0.376 | -1.365 | 0.622 | 0.127 | 0.026 | 0.044 | 0.424 | 0.578 |
| canine | 0.629 | -0.178 | 0.039 | 0.153 | 0.026 | 0.036 | 0.003 | 0.003 |
| incisor | 1.226 | 1.128 | 0.583 | 0.138 | 0.206 | 0.316 | 0.212 | 0.267 |
| mandible | -0.648 | -0.400 | 0.520 | 0.088 | 0.157 | 0.376 | 0.073 | 0.144 |
| maxilla | 0.581 | 0.359 | 0.520 | 0.079 | 0.141 | 0.376 | 0.065 | 0.144 |
| surgical procedures | -0.795 | 0.621 | 0.500 | 0.112 | 0.165 | 0.311 | 0.122 | 0.190 |
| endodontic procedures | 0.319 | -0.445 | 0.023 | 0.155 | 0.006 | 0.008 | 0.014 | 0.015 |
| restorative procedures | 0.400 | -0.289 | 0.362 | 0.067 | 0.076 | 0.238 | 0.048 | 0.124 |
